# A Nucleoside/Nucleobase-Rich Extract from *Cordyceps Sinensis* Inhibits the Epithelial–Mesenchymal Transition and Protects against Renal Fibrosis in Diabetic Nephropathy

**DOI:** 10.3390/molecules24224119

**Published:** 2019-11-14

**Authors:** Zhonghua Dong, Yueyue Sun, Guangwei Wei, Siying Li, Zhongxi Zhao

**Affiliations:** 1 School of Pharmaceutical Sciences, Shandong University, 44 West Wenhua Road, Jinan 250012, China; 201514330@mail.sdu.edu.cn (Z.D.); 13021717075@163.com (Y.S.); 2 School of Basic Medical Sciences, Shandong University, 44 West Wenhua Road, Jinan 250012, China; gwwei@yahoo.com; 3 Shandong Engineering & Technology Research Center for Jujube Food and Drug, 44 West Wenhua Road, Jinan 250012, China

**Keywords:** nucleosides and nucleobases, diabetic nephropathy, HK-2 cells, extracellular matrix, epithelial–mesenchymal transition

## Abstract

*Cordyceps Sinensis*, a traditional Chinese medicine and a healthy food, has been used for the treatment of kidney disease for a long time. The aim of present study was to isolate a nucleoside/nucleobase-rich extract from *Cordyceps Sinensis* (CS-N), determine the contents of nucleosides and nucleobases, and explore its anti-diabetic nephropathy activity. CS-N was isolated and purified by using microporous resin and glucan columns and the unknown compounds were identified by using HPLC-DAD and LC-MS. The effects of CS-N on the epithelial–mesenchymal transition (EMT), extracellular matrix (ECM) depositions, and the MAPK signaling pathway were evaluated in streptozotocin (STZ)-induced diabetic mice and high glucose (HG)-exposed HK-2 cells. CS-N significantly attenuated the abnormity of renal functional parameters, ameliorated histopathological changes, and inhibited EMT and ECM accumulation by regulating p38/ERK signaling pathways. Our findings indicate that CS-N exerts a therapeutic effect on experimental diabetic renal fibrosis by mitigating the EMT and the subsequent ECM deposition with inhibition of p38 and ERK signaling pathways.

## 1. Introduction

Driven by the ageing population, increasingly sedentary lifestyle patterns, and the rising morbidity of obesity, the global prevalence of diabetes mellitus (DM) is growing at an alarming rate. Diabetic nephropathy (DN) is one of the most serious complications in diabetic patients and the primary cause of end-stage renal failure [1]. DN is characterized by the excessive deposition of extracellular matrix (ECM) proteins, such as collagen and fibronectin, in the glomerulus and renal tubulointerstitium, eventually leading to glomerulosclerosis and interstitial fibrosis [2]. Despite the enormous burden in health care costs and the extensive and growing morbidity of the disease, there is no effective cure for diabetic nephropathy [3]. 

Myofibroblasts, the principal effector cells responsible for the synthesis and secretion of ECM, are derived from resident renal fibroblasts, circulating bone marrow precursors, and the epithelial–mesenchymal transition (EMT) of resident tubuloepithelial cells [4]. The EMT of renal tubular epithelial cells is elicited in vitro under various conditions, including stimulation with high glucose (HG), TGF-β1, or advanced glycationend products (AGEs) [5,6,7]. The first two key events of the EMT process are the reduction of intercellular epithelial adhesion molecules (E/P-cadherin and/or ZO-1) and the induction of mesenchymal proteins (α-SMA and/or vimentin) [8,9].

*Cordyceps sinensis* (*Cordyceps*, Dong Chong Xia Cao), used extensively as a traditional Chinese medicine or as a healthy food, is considered an abundant source of bioactive substances. It is reported to possess various biological activities, including antiaging, antioxidant, reparative properties, anti-cancer, immuno-stimulation, and renal-protective properties [10,11]. In China, *Cordyceps sinensis* is one of the most commonly used ingredients in traditional Chinese medicine for the treatment of people with chronic kidney disease (CKD), which can be caused by diseases such as diabetes [11,12]. Studies about the effects of *Cordyceps* on DN have been done in recent years. Sung-Hsun Yu et al. reported that *Cordyceps militaris* preserves renal function in type 2 diabetic nephropathy mice [13]. Yuan Dong et al. demonstrated the protection effects of *Cordyceps militaris* extracts against diabetic nephropathy in streptozotocin (STZ)-induced diabetic rats [14]. However, the active components and pharmacological mechanisms of *Cordyceps sinensis* for DN are not fully understood.

Nucleosides and nucleobases, the major active components in *Cordyceps sinensis*, are used as valuable chemical markers for quality control in *Cordyceps* [15]. Various studies have been published in recent years investigating their biological effects and finding that the nucleosides and nucleobases possess multiple pharmacological actions, such as anti-inflammatory, anti-cancer, anti-fibrotic, and cardioprotective activities [16,17,18,19]. Adenosine is reported to play an important role in mediating dysfunctional signaling pathways such as ERKs and p38 MAPK in diabetes mellitus and associated renal complications [20,21,22]. Guanosine increases extracellular adenosine, inosine, uridine, thymidine, and cytidine levels and decreases extracellular uric acid levels, which may be associated with its protective effects against injury in various organs [23,24].

In this study, a nucleoside/nucleobase-rich extract from *Cordyceps Sinensis* (CS-N) was isolated and characterized, and its reno-therapeutic effects were determined in vivo and in vitro. A diabetic mouse model was established by STZ injection. The therapeutic effects of CS-N on diabetic nephropathy were evaluated by determination of renal function parameters, ECM deposition, and EMT markers in renal tissues. Besides, we investigated the effects of CS-N on renal tubular epithelial cells induced by high glucose. What is more, we also attempted to clarify the signal transduction pathway associated with the reno-therapeutic effects of CS-N. CS-N may be a potent agent for the therapeutic interventions in DN. This study paves a way for the application of ingredients containing nucleosides and nucleobases in the treatment of DN.

## 2. Results

### 2.1. Analysis of Nucleosides and Nucleobases in CS-N

**Figure 1:**
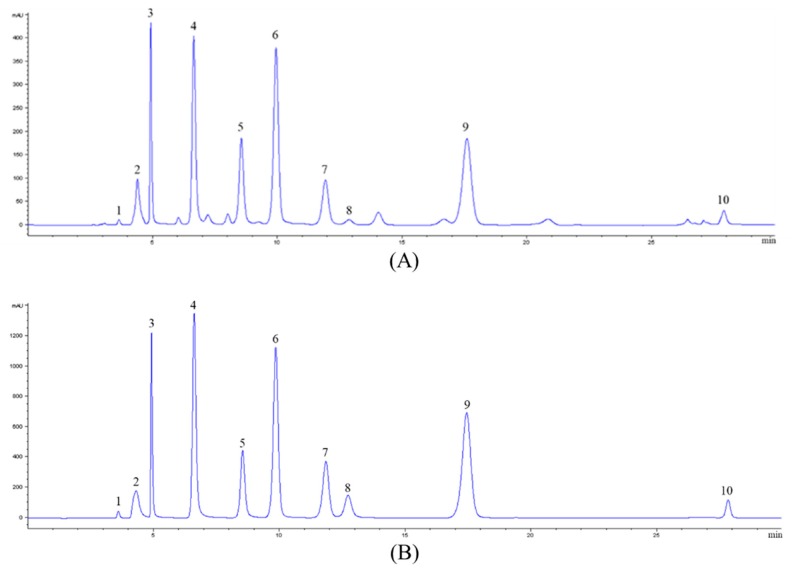
A representative HPLC chromatogram acquired at 260 nm of (**A**) nucleoside/nucleobase-rich extract obtained from *Cordyceps Sinensis* (CS-N) and (**B**) nucleoside and nucleobase standards. Peaks were tentatively identified as: 1, cytidine; 2, adenine; 3, guanine; 4, uracil; 5, hypoxanthine; 6, uridine; 7, adenosine; 8, 2′-deoxyadenosine; 9, guanosine; 10, thymidine.

The nucleosides and nucleobases in CS-N were analyzed by a high-performance liquid chromatography-diode array detector (HPLC-DAD) and a liquid chromatograph-mass spectrometer (LC-MS). A representative chromatogram of nucleoside and nucleobase standards was obtained at 260 nm (Figure 1) and the results of mass spectrometry information are shown in Table 1. By comparing retention time, UV spectra, and mass spectrometry information, we identified nucleoside and nucleobase components in CS-N.

Multiple reaction monitoring (MRM) mode in LC-MS was used to quantify nucleoside and nucleobase compounds of CS-N and the results are summarized in Table 2. Ten compounds were quantified, seven of which were nucleobases (cytidine, adenine, guanine, uracil, hypoxanthine, uridine, and thymidine) and three of which were nucleosides (2’-deoxyadenosine, guanosine, and adenosine). Among the quantified nucleoside and nucleobase compounds, guanosine was the most abundant one in CS-N, with the concentration of 194.91 ± 2.39 mg/g, and was followed by uridine with 150.70 ± 2.65 mg/g.

### 2.2. Effects of CS-N on Body Weights and Renal Function Parameters

The CS-N doses of 20, 40, 80, and 160 mg/kg/day were intragastrically administrated to mice in the preliminary experiment. It was found that the mice developed symptoms of dullness, decreased activity, and weight loss at the dosage of 160 mg/kg; the lowest dosage was less effective. Therefore, we chose 40 and 80 mg/kg/day as our evaluation doses.

Body weights were measured once a week, while fasting blood glucose levels of the experimental mice were measured at weeks 0, 1, 3, 5, and 7 after treatment with CS-N. Compared with the age-matched normal mice, body weights of DN model group were significantly reduced (*p* = 0.0001, 8 weeks). CS-N at low (40 mg/kg) or high dose (80 mg/kg) could attenuate the weight loss of the DN mice (Table 3). Meanwhile, STZ-treated mice had higher glucose levels throughout the treatment process (*p* = 0.0001, 8 weeks). The fasting blood glucose value in the CS-N-treated diabetic group was similar to the value in diabetic mice (Table 4).

**Figure 2:**
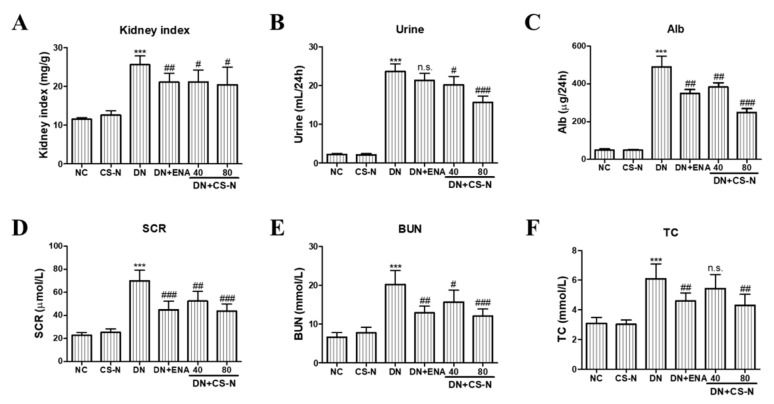
The effects of CS-N on renal functional parameters in STZ-induced diabetic mice. (**A**) Kidney index, (**B**) 24-hour urine volume, (**C**) Alb, (**D**) SCR, (**E**) BUN, and (**F**) TC. Alb: 24-hour urinary albuminuria; SCR: serum creatinine; BUN: blood urea nitrogen; TC: total cholesterol; NC: normal control; DN: diabetic nephropathy model induced by streptozotocin (STZ); ENA: positive control, 1.5 mg/kg/day enalaprilat administration; CS-N40, CS-N80: 40, 80 mg/kg/day CS-N administration. Data are presented as the means ± SDs, *n* = 6. NS-1 *p* > 0.05; *** *p* < 0.001 compared with the NC group; NS-2 *p* > 0.05; # *p* < 0.05; ## *p* < 0.01; ### *p* < 0.001 compared with the DN group.

The renal dysfunction in diabetic mice was also manifested by the elevation of kidney index and renal functional parameters, including 24-hour urine volume, 24-hour urinary albuminuria, serum creatinine (SCR), blood urea nitrogen (BUN), and total cholesterol (TC). Kidney enlargement was found in DN group (*p* = 0.0001), but the kidney index was prominently reduced by the treatment of CS-N (40 and 80 mg/kg) and enalaprilat (*p* = 0.0171, 0.0308, and 0.0063, respectively) (Figure 2A). Furthermore, the levels of 24-hour urine volume, 24-hour urinary albuminuria, SCR, BUN, and TC in diabetic mice were significantly higher than in the normal control (all *p* values were less than 0.0001) (Figure 2B–F). Eight weeks after administration of CS-N, those parameters were obviously reduced in a dose-dependent manner. In addition, enalaprilat treatment of the diabetic mice also reversed the increase of urinary albuminuria, SCR, BUN, and TC. Therefore, CS-N improved renal function in the STZ-induced diabetic mice.

### 2.3. The Effects of CS-N on Histopathological Changes in Renal Tissue

**Figure 3:**
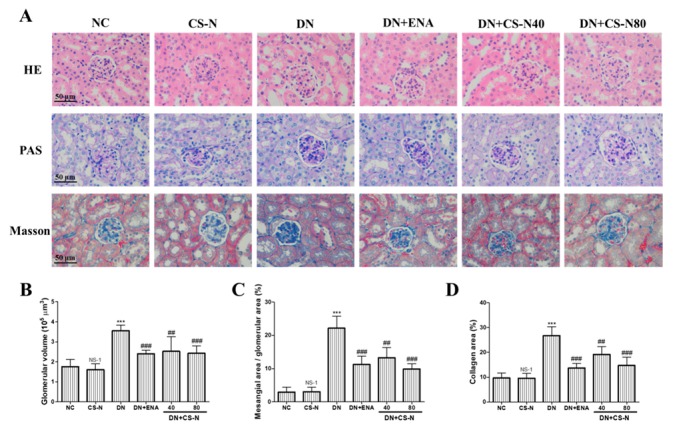
The effects of CS-N on histopathological changes in the renal tissues of STZ-induced diabetic mice. (**A**) Representative hematoxylin-eosin (HE), periodic acid-Schiff (PAS), and Masson staining of kidney sections taken from the mice of each group (*n* = 6/group) at 8 weeks. (**B**) Quantitative assessments of PAS-positive area (%) with HE staining; (**C**) Quantitative assessments of glomerular volume with PAS staining; (**D**) Quantitative assessments of Masson-positive area (%) with Masson staining. NC: normal control; DN: diabetic nephropathy; ENA: positive control, enalaprilat administration; CS-N: CS-N administration. NS-1 *p* > 0.05; *** *p* < 0.001 compared with NC; # *p* < 0.05, ## *p* < 0.01; ### *p* < 0.001 compared with DN.

We further evaluated the effects of CS-N on potential renal cortical morphological changes in the STZ-induced diabetic mice through hematoxylin-eosin (HE), periodic acid–Schiff (PAS), and Masson’s trichrome staining (Figure 3A). As shown in HE staining, there was partial tubular epithelial vacuole degeneration of renal tubules in diabetic mice. The result of Figure 3B shows obvious glomerular hypertrophy with increased glomerular volume in diabetic mice (*p* = 0.0001). Further examination of PAS and Masson’s-stained kidney tissue sections showed that the diabetic mice presented obvious mesangial matrix expansion, marked glycogen storage, and more collagen fibers in glomerular mesangium and basement membrane (Figure 3C,D). There were characteristic histological changes of glomerulosclerosis and tubulointerstitial fibrosis in the STZ-induced diabetic mice, as evidenced by mesangial matrix expansion, extracellular matrix deposition, and tubulointerstitial injury. However, administration of CS-N (especially at 80 mg/kg/day) or enalaprilat for 8 weeks significantly attenuated renal tubular vacuolar degeneration, mesangial matrix expansion, glomerulosclerosis, and collagen deposition in DN mice. 

### 2.4. The Effects of CS-N on EMT and ECM Accumulation

**Figure 4:**
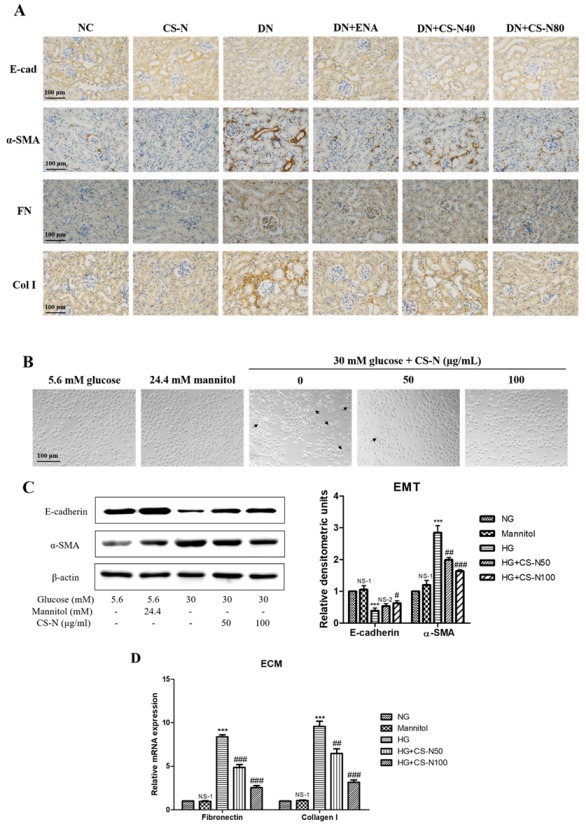
CS-N reversed epithelial-mesenchymal transition (EMT) and extracellular matrix (ECM) accumulation in STZ-induced diabetic mice and high glucose-induced HK-2 cells. (**A**) Representative renal sections with immunohistochemical staining of E-cadherin (E-cad), α-smooth muscle actin (α-SMA), fibronectin (FN), and collagen I (Col I). (**B**) Morphological changes of HK-2 cells cultured under different conditions. (**C**) Expressions of E-cadherin and α-SMA in HK-2 cells cultured under different conditions through western blotting. (**D**) Expressions of fibronectin and collagen I mRNA in HK-2 cells cultured under different conditions through RT-PCR. Data are presented as the means ± SDs, triplicate. NC: normal control; DN: diabetic nephropathy model induced by streptozotocin (STZ); ENA: positive control, 1.5 mg/kg/day enalaprilat administration; CS-N40, CS-N80: 40, 80 mg/kg/day CS-N administration. NG: 5.6 mM glucose; Mannitol: 5.6 mM glucose + 24.4 mM mannitol; HG: 30 mM glucose. NS-1 *p* > 0.05; ** *p* < 0.01, *** *p* < 0.001 compared with the NG group; NS-2 *p* > 0.05; # *p* < 0.05; ## *p* < 0.01; ### *p* < 0.001 compared with the HG group.

In order to examine the involvement of CS-N in EMT induced by STZ, the expression levels of epithelial marker, E-cadherin, and the mesenchymal marker alpha smooth muscle actin (α-SMA) in mice kidneys was detected by immunohistochemistry. In diabetic mouse kidneys, there was a lack of brownish staining of E-cadherin and an increase of α‑SMA (Figure 4A). We also attempted to verify whether CS-N inhibited hyperglycemia-instigated deposition of ECM components. The increased accumulation of ECM components was largely due to their excessive production in parallel with their reduced degradation. Fibronectin and collagen I expressions were used to evaluate the severity of ECM accumulation in diabetic mice. We detected fibronectin and collagen I expressions through immunohistochemistry (Figure 4A). Fibronectin and collagen I expression levels were up-regulated in the renal parenchyma of diabetic mice. Meanwhile, treatment of CS-N significantly decreased the expression of fibronectin and collagen I proteins. 

In vitro studies show similar results to those in mice. As in shown in Figure 4B, compared with normal glucose group, the morphology of HK-2 cells cultured in high glucose environment changed from cobblestone-like epithelial appearance to typical spindle-like shape of fibroblasts. HK-2 cells in the iso-osmotic mannitol (5.6 mM glucose plus 24.4 mM mannitol) did not change their morphological characterizations, implying that hyperosmolality has no effect on EMT. Noticeably, co-treatment with CS-N (50 and 100 μg/mL) could decrease the high glucose-induced cellular morphological and phenotypic changes. Western blot analysis showed that high glucose could significantly decrease the expression of E-cadherin and increase the expression of α-SMA (*p* = 0.0003 and 0.0001, respectively). However, CS-N distinctly reversed high glucose stimulated changes of the E-cadherin and α‑SMA (*p* = 0.0280 and 0.0007, respectively, for CS-N80) (Figure 4C). As in shown in Figure 4D, mRNA expressions of fibronectin and collagen I were markedly increased by high glucose (both *p* values were less than 0.0001), compared with the normal glucose control. Strikingly, CS-N obviously reduced the high glucose-induced increase in mRNA expressions of fibronectin and collagen I in a dose-dependent manner (both *p* values were less than 0.0001, for CS-N80). 

### 2.5. CS-N Inhibited EMT and ECM Accumulation through p38 and ERK MAPK Signaling Pathways

**Figure 5:**
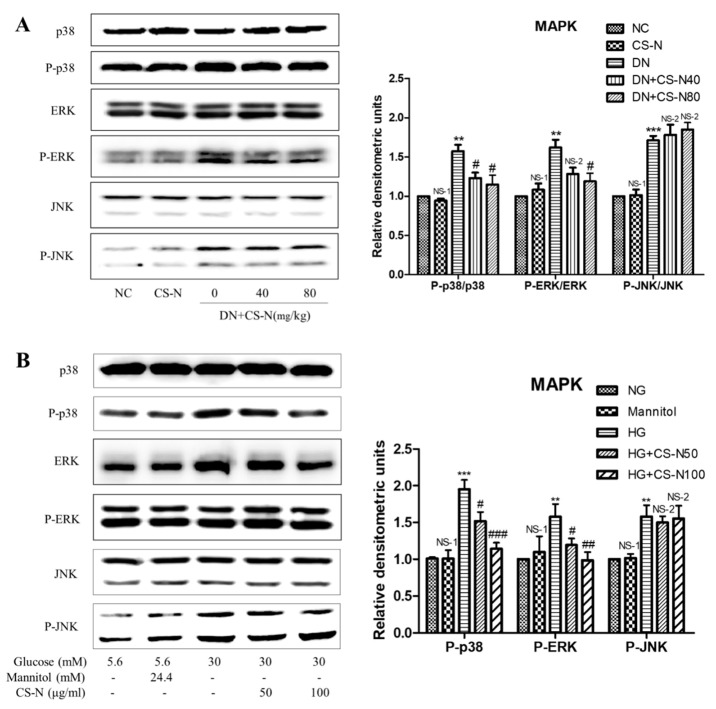
The effects of CS-N on MAPK pathway in STZ-induced diabetic mice and high glucose-induced HK-2 cells. (**A**) Expressions of p38, P-p38, ERK, P-ERK, JNK, and P-JNK in STZ-induced diabetic mice (data are presented as the means ± SDs, *n* = 3) through western blotting. (**B**) Expressions of p38, P-p38, ERK, P-ERK, JNK, and P-JNK in high glucose-induced HK-2 cells (data are presented as the means ± SDs, triplicate) through western blotting. NC: normal control; DN: diabetic nephropathy model induced by streptozotocin (STZ); CS-N40, CS-N80: 40, 80 mg/kg/day CS-N administration. NG: 5.6 mM glucose; Mannitol: 5.6 mM glucose + 24.4 mM mannitol; HG: 30 mM glucose. NS-1 *p* > 0.05; ** *p* < 0.01, *** *p* < 0.001 compared with the NC or NG group; NS-2 *p* > 0.05; # *p* < 0.05; ## *p* < 0.01; ### *p* < 0.001 compared with the DN or HG group.

The mitogen-activated protein kinase (MAPK) signaling pathway is an important modulator involved in the progression of diabetic nephropathy, so we performed western blot analysis targeting expressions of p38, ERK, and JNK. As shown in Figure 5A,B, expressions of phosphorylated p38, ERK, and JNK, significantly increased in both STZ-induced diabetic mice and high glucose-induced HK-2 cells. Treatment of normal mice with CS-N did not produce obvious changes in phosphorylated p38 or ERK expressions; however, administration of CS-N to diabetic mice significantly suppressed the phosphorylation of p38 and ERK. The treatment with CS-N did not influence the level of p-JNK in diabetic mice. Similar results were observed in CS-N-treated HK-2 cells (Figure 5B). 

**Figure 6:**
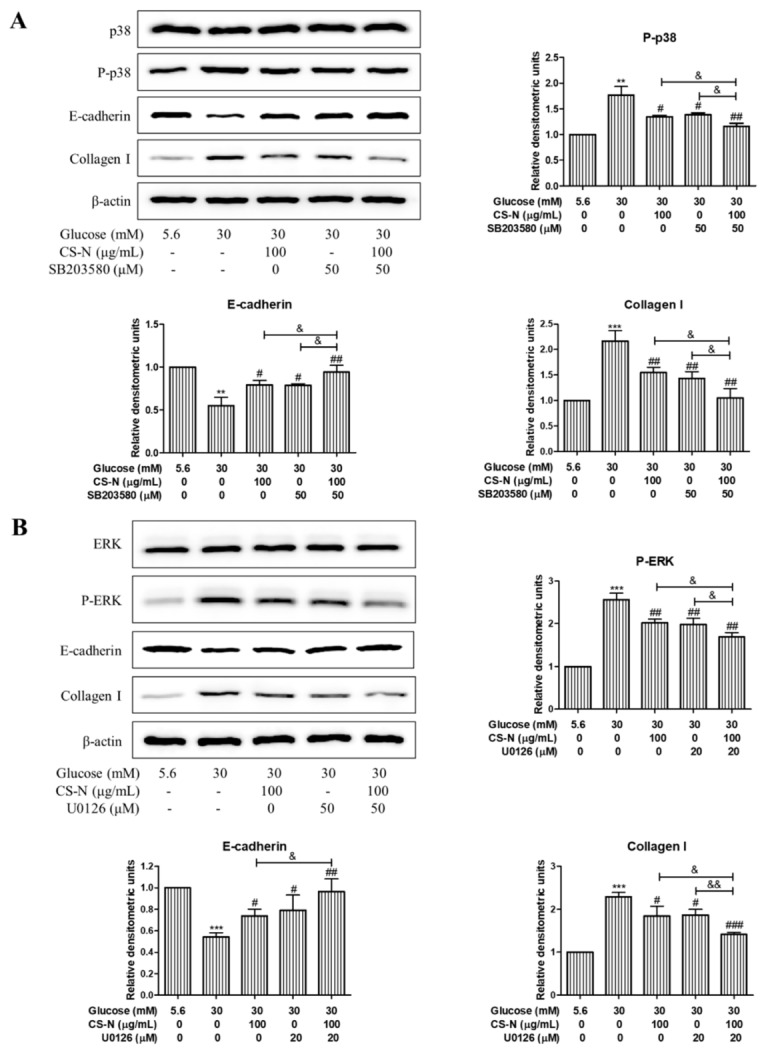
CS-N inhibits EMT and ECM accumulation in HK-2 cells via p38 and ERK MAPK pathway. (**A**) HK-2 cells were pretreated with inhibitors of p38 MAPK (SB203580, 50 μM) for 30 min, followed by the treatment with CS-N (100 μg/mL) for 48 h. Expression levels of p38, P-p38, E-cadherin, and collagen I were analyzed by western blot. (**B**) HK-2 cells were pretreated with inhibitors of ERK (U0126, 20 μM) for 30 min, followed by the treatment with CS-N (100 μg/mL) for 48 h. Expression levels of ERK, P-ERK, E-cadherin, and collagen I were analyzed by western blot. Data are presented as the means ± SDs, triplicate. ** *p* < 0.01; *** *p* < 0.001 compared with NG group; # *p* < 0.05; ## *p* < 0.01; ### *p* < 0.001 compared with HG group; & *p* < 0.05; && *p* < 0.01 compared with p38 or ERK inhibitor plus CS-N.

To further investigate the relationship between high glucose-induced HK-2 cells EMT and MAPK pathways, HK-2 cells were preincubated with the inhibitors of p38 (SB203580, 50 μM) or ERK (U0126, 20 μM) for 30 min, followed by treatment with CS-N (100 μg/mL) for 48 h. Then, the expression levels of E-cadherin and collagen I were measured by western blot. As is shown in Figure 6A, CS-N or SB203580 treatment could significantly ameliorate the high glucose stimulated changes of the E-cadherin and collagen I. Moreover, the reversing effects on E-cadherin and collagen I expression levels in cells treated with CS-N plus SB203580 were more distinct than in cells treated with CS-N or SB203580 alone. Similar results were observed in HK-2 cells treated with CS-N plus U0126 (Figure 6B). These results imply that the effects of CS-N on HK-2 cells EMT and ECM accumulation were associated with the suppression of p38 and ERK MAPK signaling. Said signaling pathways confirmed in this work are consistent with previous studies related to the adenosine and guanosine signaling in the diabetes mellitus [20,21,22,23,24].

## 3. Discussion

In this study, a nucleoside/nucleobase-rich extract was obtained from *Cordyceps Sinensis* and its nucleoside and nucleobase constituents were confirmed. Besides, we confirmed that CS-N could attenuate diabetic nephropathy by regulating p38/ERK signaling pathway. Ten nucleoside and nucleobase compounds were identified and quantified by HPLC-DAD and LC-MS analysis, seven of which were nucleobases (cytidine, adenine, guanine, uracil, hypoxanthine, uridine, and thymidine), three of which were nucleosides (2’-deoxyadenosine, guanosine and adenosine). We treated diabetic mice with CS-N for a long time and found that CS-N markedly ameliorated the increase in the levels of kidney index, 24-hour urine volume, 24-hour urinary albuminuria, SCR, BUN, and TC. The amelioration of these renal functional parameters by CS-N is associated with structural changes. As is shown in the results, CS-N could clearly inhibit DN development by inhibiting diabetes-induced abnormal kidneys, mesangial expansion, epithelial-to-mesenchymal transition, renal accumulation of glycogen, and ECM. The in vitro results showed that HK-2 cells exposed to high glucose became more elongated and less adhered, and lost their epithelial characteristics. Besides, high glucose increased ECM protein levels in HK-2 cells. However, CS-N significantly reversed all of the above changes through inhibiting the p38/ERK signaling pathway. This study separated a nucleoside/nucleobase-rich extract from *Cordyceps Sinensis* and used it to explore its therapeutic effects on diabetic nephropathy.

The current techniques for assaying nucleosides and nucleobases mainly include HPLC, ultra-performance liquid chromatography (UPLC), and capillary electrophoresis (CE) [25,26,27]. These separation techniques are frequently coupled with mass spectrometric detection, which identifies the target compounds based on the retention time (RT) and the fragmentation patterns of molecular ions [28,29]. LC–MS, through multiple reaction monitoring (MRM) mode, also contributes to the determination of nucleosides and nucleobases [30]. In this study, a macroporous resin column and glucan column were applied for the isolation and purification of nucleosides and nucleobases in *Cordyceps Sinensis*. Besides, HPLC-DAD and LC-MS analysis were successfully employed for the analyses of nucleosides and nucleobases in CS-N.

Diabetic nephropathy is featured as a progressive renal failure in combination with the augmented deposition of ECM proteins, leading to mesangial expansion, glomerulosclerosis, and atrophy [31]. From a macroscopic viewpoint, abnormal changes of renal function are mainly manifested as significant increases in kidney indexes, Alb, SCR, BUN, and TC during the development and progression of DN [32,33,34]. Tubulointerstitial fibrosis (TIF), a critical change for the progression of diabetic nephropathy to kidney failure, has been shown to be a consistent predictor of functional impairment [35]. Myofibroblasts play the foremost role in the progression of renal fibrosis in DN, while the origin of the fibroblasts remains elusive. In addition to the activation of residential fibroblasts, EMT is considered to be a direct contributor to the kidney population of myofibroblasts [36,37]. The EMT formation in tubular epithelial cells plays a significant role in TIF, which is a hallmark of DN [38,39]. Therefore, blocking renal EMT may prevent renal fibrosis. Hyperglycemia is one of the primary factors that stimulates EMT, typical of a loss of epithelial markers; acquisition of mesenchymal markers; and increased levels of ECM components in the renal cells of diabetes patients; thus, leading to DN [40,41]. Nevertheless, EMT and tubulointerstitial fibrosis in kidneys can be utilized as novel routes and potential drug targets for therapeutic interventions in DN. In the current study, we found that progressive tubulointerstitial fibrosis and renal dysfunction occurred in DN because of long-term hyperglycemia exposure. Although CS-N treatment did not alter the level of blood glucose, it showed reno-protective effects against experimental DN, as evidenced by the ameliorated renal function parameters, pathological alterations, and reversed EMT.

In the development of glomerulosclerosis and tubulointerstitial fibrosis, excessive deposition of extracellular matrix components is characterized in diabetic nephropathy [42]. Considerable evidence indicates that high glucose stimulated ECM components, including fibronectin and collagen I expressions, lead to ECM deposition, which accelerates the pathological progression of diabetic nephropathy [43,44,45]. In this study, the STZ-induced diabetic mice had increased expressions of fibronectin and collagen I in the renal tissues. Furthermore, we observed a significant downregulation of fibronectin and collagen I expressions in CS-N-treated diabetic mice. These results suggested that CS-N had a potential role in alleviating the development and progression of diabetic nephropathy through down-regulation of ECM deposition.

MAPK activation is a key modulator in the progression of renal diseases and is thought to occur in various kinds of cells, including tubular epithelial cells. Lv et al. reported that p38 MAPK may play an important role in the high glucose-induced EMT by activating AP-1 in tubular epithelial cells [35]. Wei et al. demonstrated that knockdown of thioredoxin-interacting protein ameliorates high glucose-induced EMT though regulating the p38 MAPK and ERK signaling pathways in HK-2 cells [46]. A recent study showed that JNK signaling is involved in the EMT in renal fibrosis [47]. In the present study, our data revealed that the p38, ERK, and JNK MAPK signaling pathways were activated in both HG-induced renal tubular epithelial cells and kidneys of diabetic mice. The interference of those pathways has been demonstrated to ameliorate EMT and ECM deposition in the progression of DN [48]. Importantly, our results demonstrate that the CS-N treatment significantly suppressed the phosphorylation of p38 and ERK MAPK, while the JNK phosphorylation was not affected in vivo or in vitro. Besides, we used specific inhibitors to validate whether p38 and ERK signaling pathways were involved in the inhibiting effects of CS-N on EMT and ECM accumulation. Interestingly, there was a synergy between p38 and ERK inhibitors and CS-N on suppressed, high glucose-induced EMT and ECM accumulation. Based on our results, we conclude that the suppression of ERK and p38 activation by CS-N may have a beneficial effect on delaying diabetic nephropathy progression. Our study was linked well to the adenosine and guanosine signaling in the diabetes mellitus [20,21,22,23,24], indicating that the nucleosides and nucleobases in CS-N might be those active ingredients responsible for the effects of CS-N on mouse DN.

## 4. Materials and Methods 

### 4.1. Materials

*Cordyceps sinensis* fermented powder was obtained from Hangzhou Zhongmei East China Pharmaceutical Co. (Hangzhou, China). Nucleoside and nucleobase standards (Cytidine, adenine, guanine, uracil, hypoxanthine, uridine, thymidine, 2’-deoxyadenosine, guanosine, adenosine, and inosine), streptozotocin, and enalaprilat were purchased from Aladdin Biochemical Technology CO. Ltd. (Shanghai, China). Macroporous resin D101 column and Glucan G10 column were supplied by Solarbio Technology Co. Ltd. (Beijing, China). Dulbecco’s modified Eagle’s medium (DMEM), fetal bovine serum (FBS), and penicillin–streptomycin were obtained from Gibco Invitrogen (Carlsbad, CA, USA). D-glucose and D-mannitol were provided by Sigma-Aldrich (St. Louis, MO, USA). The blood glucose meter and blood glucose test strips were purchased from F. Hoffmann-La Roche Ltd. (Basel, Switzerland). The mouse albumin ELISA kit was obtained from Bethyl Laboratories (Montgomery, TX, USA). Creatinine, urea nitrogen, and total cholesterol assay kits were provided by Nanjing Jiancheng Bioengineering Institute (Nanjing, China). α-SMA antibodies were obtained from Abcam (Cambridge, UK). E-cadherin, P-p38, p38, P-ERK, ERK, P-JNK, JNK, and β-actin antibodies were purchased from Cell Signaling Technology (MA, USA). Collagen I alpha 1 was provided by R&D system (Minneapolis, MN, USA). Goat anti-rabbit IgG were obtained from ZSGB Biotechnology Co. Ltd. (Beijing, China). SB203580 (p38 inhibitor) and U0126 (ERK inhibitor) were provided by Beyotime Biotechnology Co. Ltd. (Shanghai, China). All the other reagents used in the experiments were analytical grade.

### 4.2. Isolation and Purification of CS-N

*Cordyceps sinensis* was extracted with distilled water in an ultrasonic cleaning bath at a frequency of 100 kHz for 1 h and the extraction procedure was repeated three times. The pooled extract was centrifuged to separate it from residue and concentrated in a rotary evaporator to remove water. After lyophilization, the water extract was dissolved and loaded onto a macroporous resin D101 column (3.0 × 15 cm) and eluted with distilled water at 2.0 mL/min. Then, the effluent was collected and concentrated by rotary evaporator. After that, the concentrated effluent was further purified on a Sephadex G-10 column (2.6 × 25 cm) eluted with distilled water at 1.3 mL/min to get the CS-N.

### 4.3. Analysis of Nucleosides and Nucleobases in CS-N

Analysis of nucleosides and nucleobases in CS-N was performed by HPLC-DAD and LC-MS system.

An Agilent 1260 HPLC-DAD system (Agilent Technologies, Santa Clara, CA, USA) was used for the qualitative analysis of nucleosides and nucleobases in CS-N. A Phenomenex Synergi C12 (4.6 mm × 150 mm, 4 μm) column was employed for the separation of nucleosides and nucleobases. The mobile phase was composed of 0.1% (*v*/*v*) formic acid as eluent A and methanol as eluent B, and the gradient program was conducted as follows: 0–19 min, 5% B; 19–20 min, 5–15% B; 20–30 min, 15% B. The column temperature was set to 25 °C, the flow rate was 0.4 mL/min, and the injection volume was 10 µL. Peaks of nucleosides and nucleobases were monitored at 260 nm, and UV/vis spectra were measured over a wavelength range of 200–400 nm.

LC-MS analysis was performed using a Prominence LC-20A HPLC system (Shimadzu, Kyoto, Japan) coupled to a QTrap^®^ API-5000 LC-MS hybrid triple quadrupole/linear ion trap mass spectrometer equipped with a Turbo VTM ion source (AB SCIEX Framingham, USA). Chromatographic separation was performed using a Phenomenex Synergi C12 (4.6 mm × 150 mm, 4 μm) analytical column at a temperature of 25 °C and a flow rate of 0.4 mL/min. The mobile phase was composed of 0.1% (*v*/*v*) formic acid as eluent A and methanol as eluent B, and the gradient program was conducted as follows: 0–6 min, 5% B; 6–7 min, 5–70% B; 7–20 min, 70% B. The column was re-equilibrated for 10 min, and the overall injection duty cycle was 30 min. Q1 MS (Q1) and product ion (MS2) analyses were performed for the identification of nucleosides and nucleobases. MRM experiments were performed for quantitative analysis; inosine (137.0/269.3) was used as the internal standard (IS). The mass spectrometer was operated under the following conditions: ion mode—positive; ion spray voltage—4500 V; temperature—400 °C; curtain gas—0.10 MPa; ion source gas1—0.34 MPa; ion source gas2—0.38 MPa.

Components were identified by comparing retention time, UV spectra, and mass spectral analysis with standards and quantified by LC-MS.

Stock solutions of each external standard (ES) were prepared by dissolving appropriate amounts nucleoside and nucleobase compounds in distilled water. Stock solutions were diluted with appropriate amounts of distilled water to give solutions containing different concentrations of each standard. To each solution, an appropriate amount of IS was added to yield a final concentration of 1.5 μg/mL. Data acquisition was carried out by Analyst 1.6.2 software.

### 4.4. Animal Studies.

Male C57BL/6 mice (6–8 weeks old, weighing 18–20 g) were purchased from the Laboratory Animal Center of Shandong University (Jinan, China). They were housed in a rodent facility at a constant temperature (25 °C) with a 12 h light/dark cycle and had free access to food and water for 7 days. The experiment was carried out according to the Declaration of Helsinki, and the protocol was approved by the Animal Care and Use Committee of Shandong University (number 2016020, Jinan, Shandong, China). Then animals were induced to diabetes by intraperitoneal injection of freshly prepared STZ (dissolved in 0.01 M citrate buffer, pH 4.5) at a single dose of 130 mg/kg after a 16 h overnight fasting. Induction of the diabetes was confirmed by measuring the blood glucose levels after 3 days STZ administration. The mice with blood glucose concentrations > 16.7 mmol/L were classified successful diabetes models and used in the study. The mice were randomly divided into the following six groups (*n* = 6 per group): normal control group (NC), CS-N-control group (80 mg/kg/day, NC + CS-N), diabetic nephropathy group (DN), CS-N-treated diabetic groups (40 or 80 mg/kg/day, DN+CS-N), and enalaprilat-treated diabetic group (positive drug, 1.5 mg/kg/day, DN+ENA). Seven days after STZ injection, CS-N and enalaprilat were dissolved in distilled water and administered daily by gavage to diabetic or CS-N-control mice for 8 weeks. The mice in both control and model groups were given the same volumes of vehicle (distilled water) by gavage. Body weights were measured once a week. Blood was sampled from the tail vein at 2-week intervals, and fasting blood glucose levels were measured using a blood glucose meter.

At last, 24-hour urine samples were collected from all mice using metabolic cages. Blood samples were drawn from the orbits of all mice before they were sacrificed. Mice kidneys were rapidly excised, weighed, and cleaned in ice-cold PBS. One kidney from each mouse was fixed in 10% formaldehyde, while the other one was snap-frozen in liquid nitrogen and stored at −80 °C for subsequent testing.

### 4.5. Cell Culture and Treatment

The human proximal tubular cell line HK-2 cells (American Type Culture Collection, Manassas, VA) were cultured in DMEM (low glucose) containing 10% FBS and 1% penicillin-streptomycin at 37 °C in a humidified 5% CO_2_ atmosphere. 

The cells (HK-2) were harvested when grown to sub-confluent state and were sub-cultured in 6-well plates at a density of 1.5 × 10^5^ cells per well. HK-2 cells were cultured for 24 h in DMEM medium containing 5.6 mmol/L glucose and 0.5% FBS, and then used for experiments. The cells were treated with 5.6 mmol/L glucose (NG) and 30 mmol/L glucose (HG), and 24.4 mmol/L mannitol was added along with 5.6 mmol/L glucose as an osmotic control for 48 h. HK-2 cells cultured with 30 mmol/L glucose were co-treated with or without CS-N (50, 100 μg/mL). 

### 4.6. Renal Function Assessment

Renal function was assessed through the measurement of the kidney index, 24-hour urinary albuminuria, SCR, BUN, and TC. The kidney index was calculated according to the formula: kidney index (mg/g) = kidney weight (mg)/body weight (g). 24-hour urine was collected by metabolic cage and urine albumin concentration was measured by the mouse albumin ELISA kit. SCR, BUN, and TC levels were determined using corresponding assay kits.

### 4.7. Histological Examination of the Kidneys

Paraffin embedded kidney tissue sections (4 μm) were stained with HE, PAS, and Masson’s trichrome, and then observed under a microscope (Olympus Corporation, Tokyo, Japan) at 400× magnification. Slides were assessed by Image-Pro Plus 6.0 software (Media Cybernetics, Silver Spring, MD, USA) in a blind manner. The volume of glomeruli was calculated as follows: glomerular volume = glomerular area^1.5^ × 1.38/1.01 [49].

### 4.8. Immunohistochemical Analysis

Expressions of fibronectin, collagen I, α-SMA, and E-cadherin in the kidney tissues of mice were determined by immunohistochemistry. Paraffin-embedded kidney tissue sections were deparaffinized, rehydrated, and subjected to microwave-based antigen retrieval in citrate buffer. A blocking step was performed using 10% normal goat serum. The kidney sections were incubated overnight at 4 °C with anti-fibronectin, collagen I, α-SMA, and E-cadherin primary antibodies, and with polyperoxidase-anti-mouse IgG (ZSGB-BIO, Beijng, China). Immunoreactive signals were developed upon incubation with 3,3-diaminobenzidine (DAB, ZSGB-BIO). Images of fibronectin, collagen I, α-SMA, and E-cadherin were obtained and photographed under a microscope (Olympus Corporation, Tokyo, Japan) at 200× magnification. 

### 4.9. Morphological Observations of HK-2 Cells

HK-2 cells in different groups were washed three times with PBS and viewed under an inverted microscope (Olympus IMT-2, Tokyo, Japan). The morphology of the cells was observed under 100× magnification.

### 4.10. RT-PCR Analysis

Total RNA was extracted from HK-2 cells using TRIzol^®^ reagent (Life Technologies, Inc., Waltham, MA, USA) according to the manufacturer’s instructions and was reverse-transcribed into first strand cDNA using reverse transcriptase (Toyobo, shanghai, China). The levels of gene mRNA transcripts were analyzed by using the specific primers and SYBR Green I reagent and the reverse transcription-polymerase chain reaction (RT-PCR) kit, on Bio-Rad iQ5 Quantitative PCR System (Takara, China). Primers used for mRNA detection are listed in Table 5. Relative gene expression was quantified using the comparative threshold cycle (ΔΔ Ct) with β-actin as the endogenous multiplexed control.

### 4.11. Western Blot Analysis

Total proteins were prepared from kidneys and HK-2 cells using RIPA lysis buffer (Millipore, Bedford, MA, USA). Samples containing 30 μg total protein were loaded on 10% SDS–polyacrylamide gels and the separated proteins were transferred to polyvinylidene fluoride (PVDF) membranes. The membranes were blocked with 5% non-fat dry milk in Tris-buffered saline (TBS) for 2 h, and then probed at 4 °C overnight with primary antibodies (dilution, 1:1000), including E-cadherin, α-SMA, collagen I, P-p38, p38, P-JNK, JNK, P-ERK, ERK, and β-actin; that was followed by incubation with horseradish peroxidase-conjugated goat anti-rabbit immunoglobulin G (Santa Cruz Biotechnology, Santa Cruz, CA, USA) diluted 1:5000 in the blocking buffer for 2 h. Bound proteins were visualized with the chemiluminescence (ECL) detection system (Amersham, Little Chalfont, UK). Relative protein band density was quantified by AlphaView SA software. 

### 4.12. Statistics

The data were presented as the means ± SDs (standard deviations) of at least three experiments. Unpaired Student’s *t*-tests and ANOVA were used for the comparisons between two groups. Differences were considered significant if the *p*-values were less than 0.05.

## 5. Conclusions

In conclusion, our data demonstrate that CS-N not only attenuated diabetic nephropathy by ameliorating changes in renal pathophysiology, but suppressed the EMT and ECM accumulation of tubular epithelial cells through p38/ERK signaling pathways. Nucleosides and nucleobases may be important active components in CS-N contributing to the renal-therapeutic ability. Further investigation of the mechanistic insight into the critical role of CS-N as a promising anti-diabetic agent may be useful for diabetic nephropathy therapy.

## Figures and Tables

**Table 1:** Nucleosides and nucleobases identified by LC-MS in CS-N.

Compounds	t_R_ (min)	[M + H]^+^ (m/z)	MS2 (m/z)	DP (V)	CE (eV)
Cytidine	3.69	244.2	111.7	50	15
Adenine	4.44	136.4	118.6	50	33
Guanine	5.09	152.2	134.7	100	32
Uracil	6.98	114.0	69.3	120	27
Hypoxanthine	9.57	137.2	109.3	150	39
Uridine	11.12	245.2	112.7	60	14
2’-Deoxyadenosine	11.53	252.3	135.6	60	21
Guanosine	11.53	284.2	151.8	60	12
Adenosine	11.55	268.2	135.7	60	22
Thymidine	11.62	243.2	126.5	30	13

**Table 2:** Quantification results of nucleoside and nucleobase compounds in CS-N.

Compounds	Content (mg/g)	Compounds	Content (mg/g)
Cytidine	2.81 ± 0.21	Uridine	150.70 ± 2.65
Adenine	6.91 ± 0.32	Adenosine	41.64 ± 1.16
Guanine	95.84 ± 0.58	2’-Deoxyadenosine	13.02 ± 0.78
Uracil	74.12 ± 1.07	Guanosine	194.91 ± 2.39
Hypoxanthine	37.82 ± 1.42	Thymidine	11.89 ± 0.44

**Table 3:** The effects of CS-N on body weight (g) in STZ-induced diabetic mice (*n* = 6).

Week	NC	NC + CS-N	DN	DN + ENA	DN + CS-N40	DN + CS-N80
**0**	22.02 ± 1.14	21.42 ± 0.85	21.62 ± 0.94	21.6 ± 1.56	21.28 ± 0.76	21.63 ± 0.84
**1**	23.54 ± 1.14	22.65 ± 0.81	18.84 ± 1.23 *	18.91 ± 1.64	18.40 ± 1.39	19.17 ± 2.02
**2**	24.24 ± 1.36	23.67 ± 0.93	18.55 ± 1.89 *	19.84 ± 1.55	19.21 ± 1.42	19.97 ± 2.09
**3**	25.64 ± 1.70	24.37 ± 1.34	18.33 ± 2.12 *	20.63 ± 1.48	19.96 ± 1.31	20.40 ± 2.10
**4**	26.22 ± 1.53	25.07 ± 1.39	18.41 ± 2.48 *	20.38 ± 1.51	19.92 ± 1.52	21.07 ± 1.47 ^#^
**5**	26.69 ± 1.55	25.67 ± 1.58	18.73 ± 2.25 *	20.72 ± 1.39	20.72 ± 1.54	21.28 ± 1.65 ^#^
**6**	27.09 ± 2.23	26.13 ± 1.45	17.39 ± 2.44 *	20.28 ± 2.12	20.43 ± 1.36 ^#^	21.17 ± 2.54 ^#^
**7**	27.88 ± 2.22	26.57 ± 1.40	17.13 ± 2.05 *	20.61 ± 2.08	20.42 ± 1.82 ^#^	20.73 ± 2.50 ^#^
**8**	28.38 ± 2.27	26.97 ± 1.78	17.27 ± 1.68 *	20.33 ± 2.17 ^#^	20.59 ± 1.83 ^#^	20.83 ± 2.53 ^#^

NC: normal control; DN: diabetic nephropathy model induced by streptozotocin (STZ); ENA: positive control, 1.5 mg/kg/day enalaprilat administration; CS-N40, CS-N80: 40, 80 mg/kg/day CS-N administration. * *p* < 0.05 compared with NC; # *p* < 0.05 compared with DN.

**Table 4:** The effects of CS-N on fasting blood glucose (mmol/L) in STZ-induced diabetic mice (*n* = 6).

Week	NC	NC + CS-N	DN	DN + ENA	DN + CS-N40	DN + CS-N80
**1**	6.15 ± 0.38	5.80 ± 0.75	26.03 ± 2.37 *	26.77 ± 1.90	27.20 ± 2.47	25.80 ± 3.06
**3**	6.20 ± 0.24	6.05 ± 0.55	27.82 ± 1.52 *	27.27 ± 1.65	27.17 ± 2.29	27.78 ± 1.87
**5**	5.83 ± 0.36	6.07 ± 0.58	27.62 ± 1.67 *	26.97 ± 1.87	26.05 ± 2.67	27.00 ± 1.06
**7**	6.03 ± 0.52	6.13 ± 0.45	27.50 ± 0.95 *	26.30 ± 0.92	26.80 ± 1.34	26.73 ± 1.06

NC: normal control; DN: diabetic nephropathy model induced by streptozotocin (STZ); ENA: positive control, 1.5 mg/kg/day enalaprilat administration; CS-N40, CS-N80: 40, 80 mg/kg/day CS-N administration. * *p* < 0.05 compared with NC.

**Table 5:** Primer sequences used in real-time PCR analysis.

Name	Accession Number	Primer
Fibronectin	NM_212482.3	Forward: 5′-TGGAGGAAGCCGAGGTTT-3′ Reverse: 5′-CAGCGGTTTGCGATGGTA-3′
Collagen I	NM_000088.3	Forward: 5′-GTGCGATGACGTGATCTGT-3′ Reverse: 5′-TTGGTCGGTGGGTGACTCT-3′
β-actin	NM_001101.5	Forward: 5′-ACTCTTCCAGCCTTCCTTCC-3′ Reverse: 5′-GAGGAGCAATGATCTTGATCTTC-3′
